# Investigation of Intraoperative Bacterial Contamination in Surgical Instruments Used for Total Hip Arthroplasty

**DOI:** 10.1016/j.artd.2026.102027

**Published:** 2026-04-30

**Authors:** Akihito Oya, Noboru Matsumura, Atsuhiro Fujie, Shu Kobayashi, Kengo Harato, Mitsuru Yagi, Taku Suzuki, Robert Nakayama, Arihiko Kanaji, Morio Matsumoto, Masaya Nakamura

**Affiliations:** a Department of Orthopedic Surgery, Keio University School of Medicine, Tokyo, Japan; b Keio Orthopedic Advancing Squad for the Interactive Study(OASIS), Keio University School of Medicine, Tokyo, Japan; c Sports Medicine Research Center, Keio University, Yokohama, Kanagawa, Japan; d Department of Orthopaedic Surgery, International University of Health and Welfare School of Medicine, Narita, Chiba, Japan; e Department of Orthopedic Surgery Restorative Medicine of Neuro-Musculoskeletal System, Fujita Health University School of Medicine, Aichi, Nagoya, Japan

**Keywords:** Total hip arthroplasty, Instrument contamination, Femoral broaches, Periprosthetic joint infection

## Abstract

**Background:**

Reusing surgical instruments during simultaneous bilateral total hip arthroplasty (THA) may reduce direct costs but may increase the risk of periprosthetic joint infections, particularly through femoral broaches used for canal preparation. This study investigated potential bacterial contamination of surgical instruments to assess the safety of their reuse.

**Methods:**

Seventy-eight primary THA cases (15 males, 63 females; mean age, 63.8 ± 11.2 years) performed between January 2021 and October 2022 were analyzed. This prospective study evaluated bacterial contamination of the femoral broach during THA. In cases of simultaneous bilateral procedures, sampling was conducted after completion of the first-operated side. Patients were categorized according to operative duration (<90 minutes vs ≥ 90 minutes) using a predefined cutoff based on previous literature. Additional comparisons were made between culture-positive and culture-negative groups.

**Results:**

Bacterial contamination was detected in 3 cases (3.8%), all involving skin commensals (*Staphylococcus capitis* or *Micrococcus luteus*), with no periprosthetic joint infections reported. All positive cultures occurred in the short-time group. Operative time showed no association with contamination, whereas body weight and body mass index were significantly higher among culture-positive patients.

**Conclusions:**

Although no clinically significant infections occurred, bacterial contamination of surgery instruments was observed. Reusing a single instrument set may be safe with strict infection control measures; however, additional vigilance is warranted for obese patients due to their higher contamination risk.

## Introduction

Total hip arthroplasty (THA) is a widely performed and highly effective surgical procedure for managing hip joint disorders [1]. Continuous advancements in surgical techniques and perioperative care have improved clinical outcomes [2,3]. However, THA requires specialized instruments for both acetabular and femoral preparation, and single-use practices significantly increase procedural costs. Recently, simultaneous bilateral THA has become more common, offering advantages such as shorter hospital stays, lower overall healthcare costs, and faster postoperative recovery [4,5]. In clinical practice, simultaneous bilateral THA is sometimes performed by a single surgical team during one anesthetic session. In this situation, surgical instruments used for the first hip may be reused for the contralateral side, which raises concerns regarding potential bacterial contamination and instrument-related transmission.

Although reusing one instrument set can enhance procedural efficiency and reduce expenses, it may also increase the risk of periprosthetic joint infection (PJI) through intraoperative contamination. The estimated cost was calculated based on institutional charges and included personnel expenses (approximately 30 USD), instrument rental and sterilization costs (approximately 26 USD), and a minimum transportation cost (approximately 83 USD), for a total of approximately 140 USD. These values reflect the cost structure at our institution and may vary among healthcare systems.

Despite its clinical and economic significance, there is no established consensus on the optimal number of instrument sets required for simultaneous bilateral THA. Therefore, this study aimed to evaluate the safety of reusing a single instrument set by analyzing bacterial contamination of femoral broaches at the conclusion of primary THA procedures. We hypothesized that prolonged surgical time would lead to bacterial contamination on the broaches.

The femoral broach was selected because it frequently contacts the wound edge and surrounding skin during femoral preparation, especially in minimally invasive THA. Furthermore, this instrument is commonly reused during simultaneous bilateral procedures, which may increase the risk of bacterial transmission.

## Material and methods

### Participants

This study was approved by the Institutional Ethics Committee (approval no. 20200152). Informed consent was obtained from all participants. The study included 78 consecutive patients (15 males, 63 females; mean age, 63.8 ± 11.2 years) who underwent primary THA at our institution between January 2021 and October 2022.

### Surgical procedure

All procedures were performed by a single attending surgeon, assisted by a surgical assistant with 7-10 years of experience and an orthopaedic resident with 2-3 years of postgraduate training. Operations were performed in standard operating rooms not in bioclean environments. The use of space suits (body exhaust suits) was optional and left to the surgical team’s discretion. All surgical staff wore double gloves. Although the junction between the gown and gloves was not sealed with tape, gown sleeves extended to the mid-palm, and gloves were pulled proximally over the wrists to maintain overlap and minimize contamination. Gloves were replaced twice—once before acetabular component insertion and again before femoral stem insertion—to reduce exposure to sweat or skin contact.

Standardized surgical site preparation included skin disinfection with povidone–iodine solution followed by the application of iodine-impregnated adhesive drapes. Prophylactic antibiotics (cefazolin sodium 1 g) were administered intravenously before incision and continued twice daily (morning and evening) until postoperative day 1.

An anterolateral approach was employed with patients placed in the lateral decubitus position. The femoral stems used included Taperloc Complete Microplasty (Zimmer Biomet, Warsaw, IN, USA), Triloc (DePuy Synthes, Warsaw, IN, USA), and Corail (DePuy Synthes, Warsaw, IN, USA). Intraoperative irrigation was routinely performed at 3 stages: before acetabular cup implantation, before femoral stem insertion, and prior to wound closure. Pulse lavage using sterile saline solution was performed, with a total irrigation volume of approximately 3000 mL in each case.

### Sample collection

**Figure 1:**
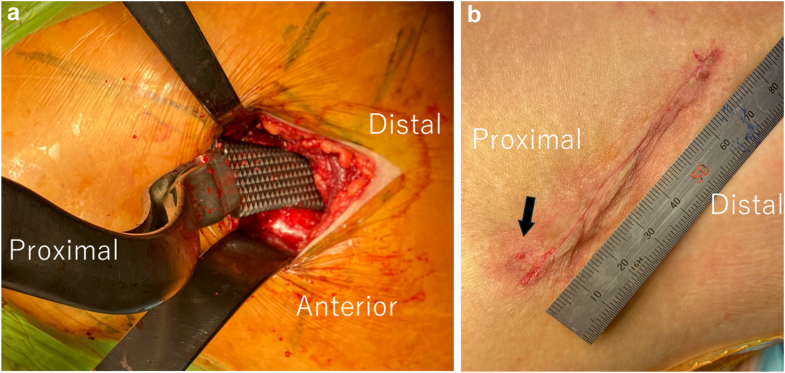
Intraoperative photographs demonstrating limited surgical exposure during femoral preparation. (a) Broaching through a small skin incision. (b) Postoperative photograph showing the incision length and minor skin abrasions (black arrow).

THA was performed through a relatively small skin incision (approximately 6-8 cm) at our institution. Because femoral exposure can be technically demanding in this minimally invasive approach, close proximity or contact between the femoral broach and the wound edge or surrounding skin may occur during femoral preparation. The intraoperative surgical field and the potential contact between the femoral broach and the surrounding skin are illustrated in Figure 1.

At the end of each procedure, the surface of the broach used for femoral canal preparation was swabbed approximately 10 times for microbiological evaluation. Sampling was performed immediately after completion of the first hip operation to simulate the clinical scenario of simultaneous bilateral THA, in which surgical instruments may be reused for the contralateral side. In cases of simultaneous bilateral procedures, samples were obtained only after completion of the first-operated side to standardize the sampling conditions.

**Figure 2:**
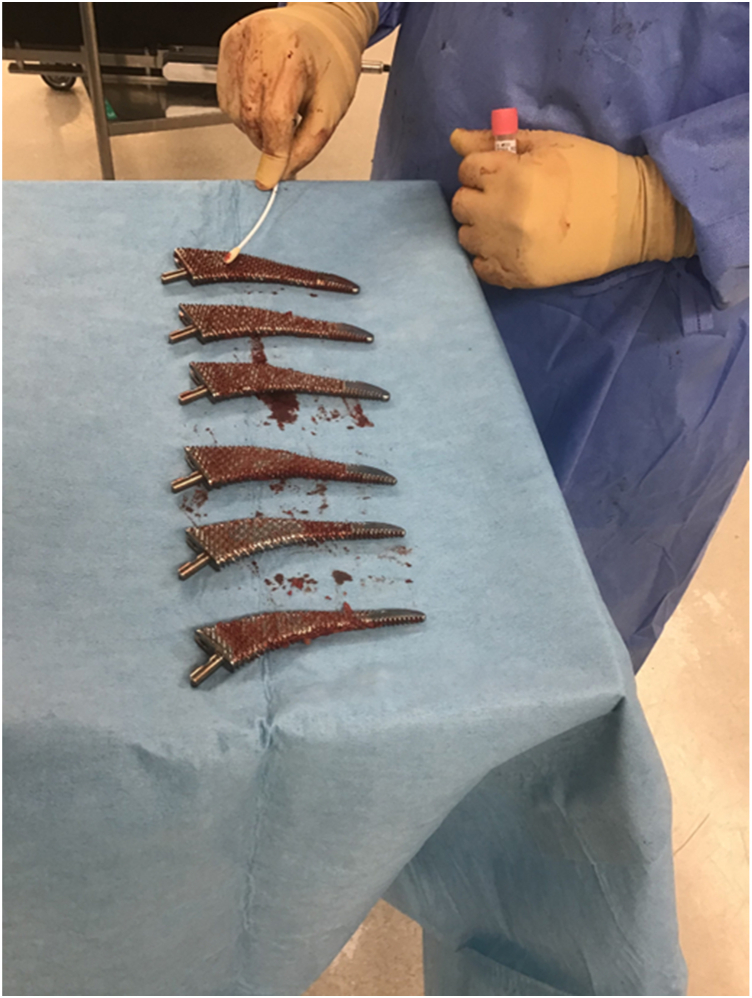
Swabbing the femoral broach to collect specimens for bacterial culture.

Each sample was collected using sterile gloves, placed in a sterile tube, and sent to the microbiology laboratory for bacterial culture, including anaerobic culture. In each case, the largest broach was selected for sampling because it was considered most likely to have contacted the skin. The swabbing procedure is illustrated in Figure 2.

### Data analysis

Patients were categorized into 2 groups based on operative time: short-time (≤89 minutes, S group) and long-time (≥90 minutes, L group). This threshold was selected a priori because prolonged operative time has been reported to be associated with an increased risk of postoperative infection after THA. Previous studies have shown that operative times exceeding 90 minutes are associated with a higher risk of infection, with incremental increases in deep infection risk for each additional 10 minutes of operative time [6]. Baseline characteristics were compared between the 2 groups using Student’s *t*-test or the chi-square test, as appropriate. The chi-square test was also used to assess differences in bacterial culture positivity between groups. To identify factors associated with contamination, patients were further divided into culture-positive and culture-negative groups. Demographic and intraoperative variables were compared using Student’s *t*-test. To examine potential differences in contamination rates among implant types (Taperloc Complete Microplasty, Triloc, and Corail), Fisher’s exact test was applied due to small number sizes in some subgroups. A *P* value < .05 was considered statistically significant.

## Results

The overall mean operative time was 86.0 ± 20.9 minutes (range, 57-194 minutes). Patients were categorized based on operative time: the S group had a mean operative time of 76.8 ± 9.1 minutes (range, 57-89 minutes), whereas the L group had a mean operative time of 108.2 ± 24.5 minutes (range, 90-194 minutes). The difference in operative time between the 2 groups was statistically significant (*P* < .001). No significant differences in sex, height, weight, or body mass index (BMI) were observed between the 2 groups. The mean age of the participants was 63.8 ± 11.2 years (range, 32-88 years), with mean ages of 64.6 ± 10.4 years (range, 32-88 years) in the S group and 61.7 ± 13.0 years (range, 39-78 years) in the L group. There was no significant difference between the groups. The overall mean intraoperative blood loss was 170.6 ± 116.3 mL (range, 10-610 mL). The mean blood loss was 141.7 ± 81.8 mL (range, 10-440 mL) in the S group and 239.8 ± 154.4 mL (range, 40-610 mL) in the L group, indicating significantly greater blood loss in the L group (*P* < .001). Table 1 summarizes patient characteristics for both groups.

**Table 1:** Patient background by operative time group.

Parameter	Overall	Short-time group (S)	Long-time group (L)	*P* value
Age (y)	63.8 ± 11.2 (32-88)	64.6 ± 10.4 (32-88)	61.7 ± 13.0 (39-78)	.297
Height (cm)	158.4 ± 9.0 (132.9-188.0)	158.2 ± 8.5 (132.9-179.0)	159.0 ± 10.2 (149.9-188.0)	.695
Weight (kg)	59.0 ± 13.6 (37.5-101.8)	58.9 ± 12.9 (39.3-101.8)	59.4 ± 15.3 (37.5-93.6)	.812
BMI (kg/m^2^)	23.4 ± 4.6 (14.5-40.0)	23.5 ± 4.6 (14.5-40.0)	23.3 ± 4.6 (15.7-37.5)	.842
Operative time (min)	86.0 ± 20.9 (57-194)	76.8 ± 9.1 (57-89)	108.2 ± 24.5 (90-194)	<.001^a^
Blood loss (mL)	170.6 ± 116.3 (10-610)	141.7 ± 81.8 (10-440)	239.8 ± 154.4 (40-610)	<.001^a^
Implant system (Taperloc Complete Microplasty/Triloc/Corail)	65 / 11 / 2	50 / 5 / 0	15 / 6 / 2	

a *P* < .001.

Bacterial contamination was detected in 3 cases (3.8%), all within the S group. The identified organisms were skin commensals—*Staphylococcus capitis* and *Micrococcus luteus*—and none of these cases developed PJI (Table 2).

**Table 2:** Positive culture cases (in the short-time group).

Case	Age	Sex	Height (cm)	Weight (kg)	BMI (kg/m^2^)	Operative time (min)	Blood loss (mL)	Stem type	Detected bacteria
1	52.0	F	159.6	101.8	40.0	82.0	225.0	Taperloc Complete Microplasty	*Micrococcus luteus*
2	57.0	F	159.0	89.2	35.3	77.0	185.0	Taperloc Complete Microplasty	*Staphylococcus epidermidis*
3	58.0	F	155.0	47.7	19.9	71.0	10.0	Triloc	*Staphylococcus capitis*
Mean	55.7		157.9	79.6	31.7	76.7	140.0		

Contrary to expectations, all positive cultures were observed in the S group, suggesting that longer operative time does not necessarily increase the risk of instrument contamination. To further explore contributing factors, patients were divided into culture-positive and culture-negative groups. As shown in Table 3, no significant differences were found between the 2 groups regarding age (55.7 ± 3.2 vs 64.1 ± 11.3 years, *P* = .205), intraoperative blood loss (140.0 ± 114.3 vs 171.9 ± 117.0 mL, *P* = .645), or operative time (76.7 ± 5.5 vs 86.4 ± 21.2 minutes, *P* = .433). No significant difference in contamination rates was observed among the 3 implant systems (*P* = .379 [Taperloc Complete Microplasty vs Triloc]; *P* = 1.000 [Taperloc Complete Microplasty vs Corail]; *P* = 1.000 [Triloc vs Corail], Fisher’s exact test). However, the culture-positive group had significantly higher body weight (79.6 ± 28.3 vs 58.2 ± 12.3 kg, *P* = .007) and BMI (31.7 ± 10.5 vs 23.1 ± 3.98 kg/m^2^, *P* = .001) than the culture-negative group.

**Table 3:** Comparison between the culture-positive and culture-negative groups.

Parameter	Culture-positive group	Culture-negative group	*P* value
Age (y)	55.7 ± 3.2 (52-58)	64.1 ± 11.3 (32-88)	.205
Height (cm)	157.9 ± 2.5 (155.0-159.6)	158.4 ± 9.1 (132.9-188.0)	.913
Weight (kg)	79.6 ± 28.3 (47.7-101.8)	58.2 ± 12.3 (37.5-93.6)	.007^a^
BMI (kg/m^2^)	31.7 ± 10.5 (19.8-40.0)	23.1 ± 4.0 (14.5-37.5)	.001^a^
Operative time (min)	76.7 ± 5.5 (71-82)	86.4 ± 21.2 (57-194)	.433
Blood loss (mL)	140.0 ± 114.3 (10-225)	171.9 ± 117.0 (10-610)	.645
Implant system (TCM/Triloc/Corail)	2 / 1 / 0	63 / 10 / 2	TCM vs Triloc; .379 TCM vs Corail; 1.000 Triloc vs Corail; 1.000

TCM, Taperloc Complete Microplasty.

a *P* < .01.

## Discussion

Although no previous studies have specifically recommended using 2 separate instrument sets for simultaneous bilateral THA, it is generally accepted that using newly opened sterile instruments for each side minimizes contamination and infection risk. Tateiwa et al. demonstrated that airborne particles and microorganisms in the operating room significantly increase during patient repositioning, suggesting that opening a new sterile set after repositioning may further reduce contamination risk [7]. However, preparing 2 full surgical instrument sets incurs additional costs, including rental, sterilization, transport, and labor. Boktor et al. reported a sterilization cost of £215.36 for THA when 4 trays were used [8]. To assess whether reusing a single instrument set could be a safe and cost-effective alternative, this study investigated bacterial contamination of femoral broaches at the end of each procedure.

Previous studies have reported that surgical instrument contamination risk increases with operative time; however, Uzun et al. noted that covering instruments can mitigate this effect [9]. Similarly, Warren et al. found that bacterial contamination of suction tips increased over time in a controlled laboratory setting [10]. Contrary to these findings, all positive cultures in our study occurred in the S group, with no significant difference in operative duration between culture-positive and culture-negative cases. These results suggest that under current surgical and sterilization protocols, operative time is not a primary factor influencing instrument contamination.

Notably, patients in the culture-positive group had significantly higher body weight and BMI than those in the culture-negative one, indicating a potential association between body habitus and contamination risk. Obesity may increase skin flora density or complicate the maintenance of a sterile field. Kreouzi et al. [11] suggested that obesity-induced changes in sebaceous lipid composition, specifically higher saturated fatty acid levels, could promote the growth of pathogenic *Cutibacterium acnes* strains. Furthermore, abundant subcutaneous tissue in obese patients may increase the likelihood of inadvertent contact between surgical instruments and the skin, raising the risk of contamination. Although causation cannot be confirmed due to the small sample size and low contamination rate, these findings highlight the need for further investigation and suggest that stricter infection control measures are warranted in high-BMI patients.

The bacterial species identified in this study—*Staphylococcus epidermidis*, *S capitis*, and *M luteus*—are common skin commensals. Considering that all surgical staff followed standard aseptic protocols, including proper hand scrubbing, double gloving, and glove changes before implant handling, these organisms most likely originated from patient skin. Nevertheless, contamination from exposed body areas of the surgical team (eg, head, face, or neck) cannot be entirely excluded. To minimize contamination, surgical instruments should avoid direct contact with the patient’s skin, and damaged iodine-impregnated drapes should be promptly replaced [12]. Because glove perforation can allow skin flora transfer, using double gloves and periodically replacing the outer pair is also recommended [13,14].

**Figure 3:**
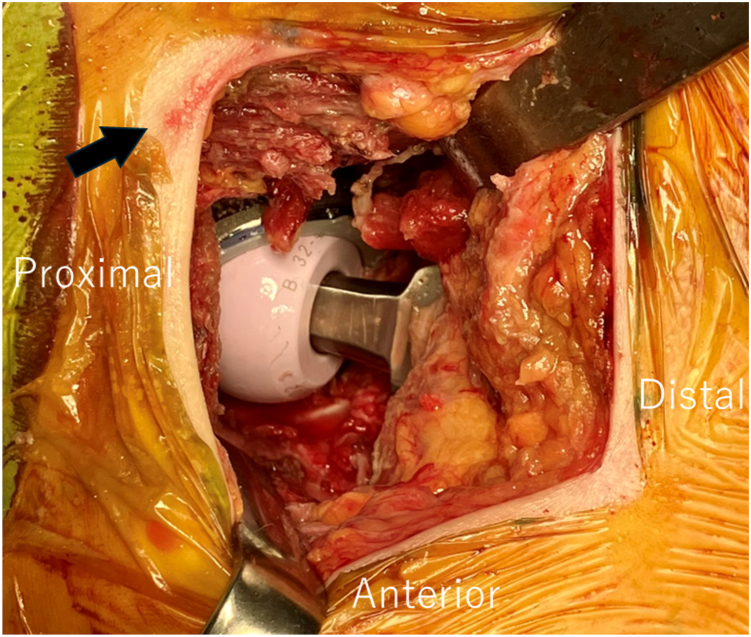
Intraoperative findings demonstrating disruption of the iodine-impregnated adhesive drape and minor skin abrasions during femoral preparation (black arrow).

The potential mechanism of contamination may be related to limited surgical exposure in minimally invasive approaches. During femoral preparation, disruption of the adhesive drape and minor skin abrasions are frequently observed in routine clinical practice, which may increase the risk of bacterial contamination of the femoral broach (Fig. 3). Therefore, surgeons should be aware of the potential risk of contamination, especially when the broach is suspected to have contacted the skin or when the sterile barrier is compromised. In such situations, decontamination or replacement of the instrument should be considered to minimize the risk of surgical site infection.

No significant differences in contamination rates were observed among the 3 implant systems (Taperloc Complete Microplasty, Triloc, and Corail). However, due to the limited number of cases, particularly with the Corail implant, the statistical power of this comparison was restricted. Larger cohort studies are needed to validate these observations.

These findings have practical implications for simultaneous bilateral THA, where reusing a single instrument set can improve efficiency and reduce costs. Although no clinical infections occurred in this cohort, strict adherence to infection control protocols remains essential. Future studies should confirm these results in larger populations and determine whether culture-positive instruments could serve as predictors for subclinical contamination or indicators of PJI. Considering the potential association between obesity and contamination, preparing 2 separate sterile instrument sets for high-BMI patients may be advisable to minimize the risk of cross-contamination during bilateral procedures.

Several limitations should be acknowledged. First, the sample size was relatively small, particularly for subgroup analyses. The low contamination rate observed in this study resulted in a limited number of culture-positive cases, which reduced the statistical power for subgroup analyses. In particular, the association between obesity and contamination should be interpreted with caution. Larger prospective studies are needed to further evaluate potential risk factors. Second, this study assessed only femoral broach surface contamination and did not examine other instruments or intraoperative factors such as glove replacement frequency or drape integrity. Third, the absence of clinical infections limits the ability to directly correlate culture positivity with PJI risk. Finally, this study was conducted by a single surgeon at a single institution with specific perioperative infection prevention protocols. Therefore, the results may not be generalizable to other surgeons or institutions. In addition, contamination of other surgical instruments, such as handles, trays, and reamers, was not evaluated. Consequently, the present findings cannot be directly applied to the reuse of entire instrument sets. Despite these limitations, this study provides valuable insights into the potential safety of reusing instruments in primary THA. Although the association between culture-positive instruments and the development of PJI remains unclear, it is nevertheless desirable to maintain instruments in an uncontaminated state. Therefore, the findings of this study remain meaningful in the context of infection control.

In conclusion, femoral broach contamination in primary THA appears to be rare and not related to operative time. Higher body weight and BMI may be associated with an increased risk of contamination, warranting further investigation to clarify their clinical significance.

## Conclusions

This study demonstrated that bacterial contamination of femoral broaches during primary THA was infrequent and did not result in PJI. These results suggest that reusing a single instrument set in simultaneous bilateral THA is safe when stringent infection control protocols are maintained. Although operative time was not associated with contamination, higher body weight and BMI were associated with increased risk. Further studies are warranted to confirm these findings and to develop safe, cost-effective guidelines for instrument reuse, particularly in obese patients.

## CRediT authorship contribution statement

**Akihito Oya:** Writing – original draft, Methodology, Investigation, Formal analysis, Conceptualization. **Noboru Matsumura:** Writing – review & editing, Writing – original draft, Project administration, Methodology, Investigation, Conceptualization. **Atsuhiro Fujie:** Investigation. **Shu Kobayashi:** Investigation. **Kengo Harato:** Writing – review & editing, Writing – original draft, Project administration, Methodology, Investigation, Conceptualization. **Mitsuru Yagi:** Writing – review & editing. **Taku Suzuki:** Writing – review & editing. **Robert Nakayama:** Writing – review & editing. **Arihiko Kanaji:** Writing – review & editing. **Morio Matsumoto:** Supervision. **Masaya Nakamura:** Supervision.

## Conflicts of interest

The authors declare there are no conflicts of interest.

For full disclosure statements refer to https://doi.org/10.1016/j.artd.2026.102027.

## Appendix A Supplementary data

Conflict of Interest Statement for All Authors
